# Supplementary material for: Dysregulation of cystathionine γ‐lyase promotes prostate cancer progression and metastasis
Source: EMBO Rep. 2019 Aug 29;20(10):e45986. doi: 10.15252/embr.201845986 (PMC6776913; doi:10.15252/embr.201845986)
Supplement: Supplementary file 1 — Appendix [file EMBR-20-e45986-s001.pdf]

Appendix

Dysregulation of cystathionine γ-lyase promotes prostate cancer progression and metastasis

Yi-Hsiang Wang* [1, 2], Jo-Ting Huang* [1], Wen-Ling Chen[1], Rrong-Hsuan Wang[3] Ming-Chien Kao[3], Yan-Ru Pan[3], Shih-Hsuan Chan[1, 2, 4], Kuo-Wang Tsai[5, 6, 7], Hsing-Jien Kung[1, 8], Kai-Ti Lin[1, 3, 9], and Lu-Hai Wang[1, 4, 9]

Table of Contents:

Appendix Figure S1. PC3-B2, B3 cells exhibit higher bone metastatic potential then PC-T2, T3 cells. **2**

Appendix Figure S2. H_2S treatment rescues reduced p65 nuclear translocation by CTH knockdown. **3**

Appendix Figure S3. Prostate orthotopic implantation of PC3 cells with reduced CTH expression decreases tumor growth and distant metastasis. **4**

Appendix Figure S4. Tube formation assay by HUVEC cells co-culturing in conditional medium from PC3-B2 cells with CTH knockdown. **5**

Appendix Table S1. Primers, siRNAs, and sgRNA used in this study. **6**

Appendix Table S2. Clinical data for 469 prostate cancer cases. **7-19**

Appendix Figures

Appendix Fig S1

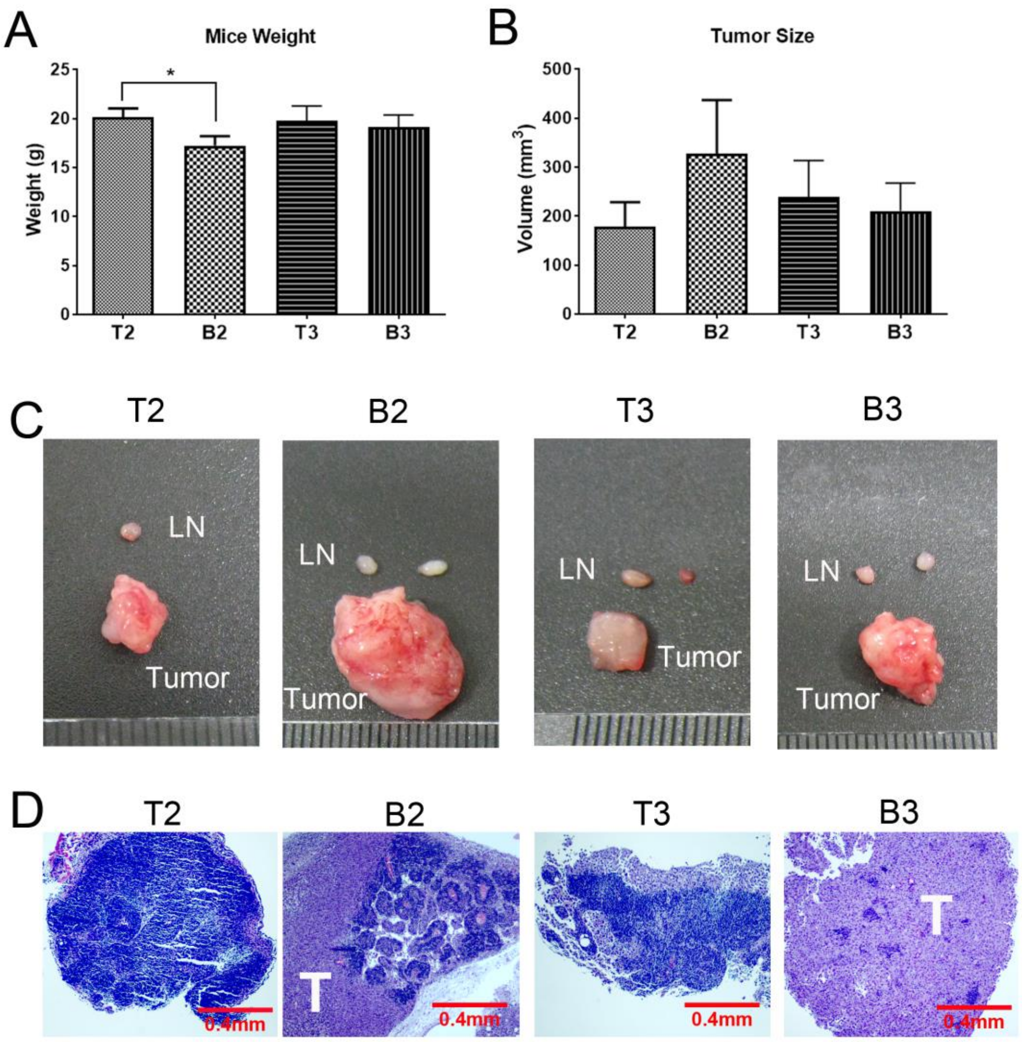

Appendix Fig S1. PC3-B2, B3 cells exhibit higher bone metastatic potential then PC-T2, T3 cells. $1x10^6$ PC3-T2, T3, B2, or B3 cells were orthotopically injected into mouse prostate for 15-20 days. (A-B) The mice weight (A) and the tumor volume (B) were compared. Data are presented as means ± SEM (n = 6-8 mice per group). Student's t-test was used for the statistical analysis (*P<0.05). (C) Images of the orthotopic xenograft tumors and paraaortic lymph nodes of mice orthotopically implanted with PC3-T2, T3, B2, or B3 cells. (D) Images show H&E staining of paraaortic lymph node sections. T: metastatic tumor cells (scale bar: 0.4mm).

Appendix Fig S2

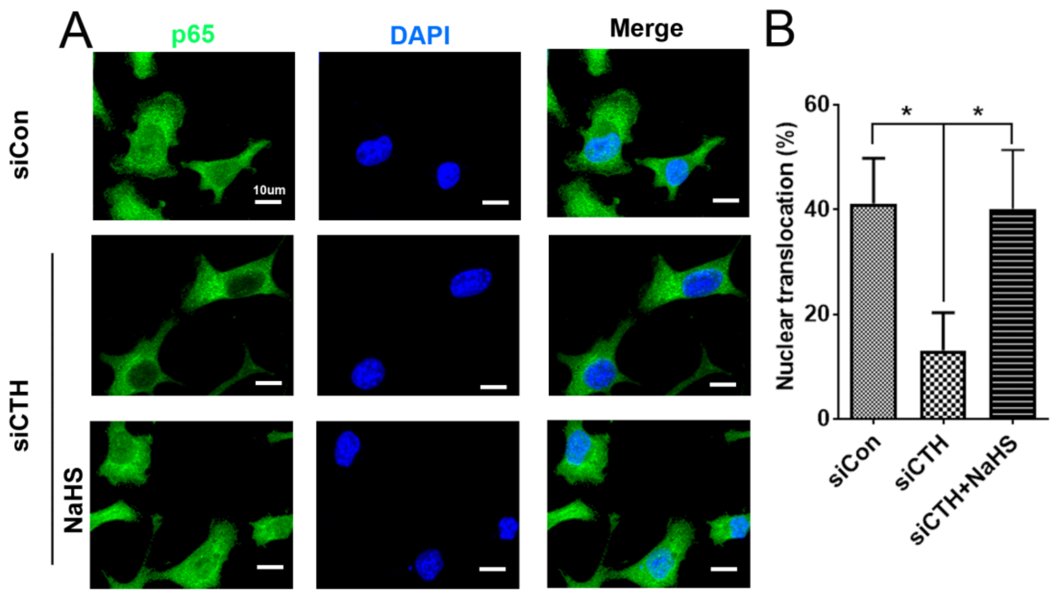

Appendix Fig S2. H_2S treatment rescues reduced p65 nuclear translocation by CTH knockdown. (A) PC3 cells transfected with control or CTH siRNA were serum-starved overnight and then treated with 10% FBS with or without NaHS (100μM) for 1hr. Subcellular localization of p65 was detected by immunocytochemistry. Nuclei were counterstained with DAPI. The representative images are shown. Scale bars: 10μM. (B) The percent of nuclear staining for p65 was scored by counting the positive-nuclear stained cells and the total number of cells quantified in random microscopic fields. Data shown represent the means ± SD (n=3 biological replicates). ANOVA followed by Tukey's post-hoc test was used for the statistical analysis. (*P<0.05).

Appendix Fig S3

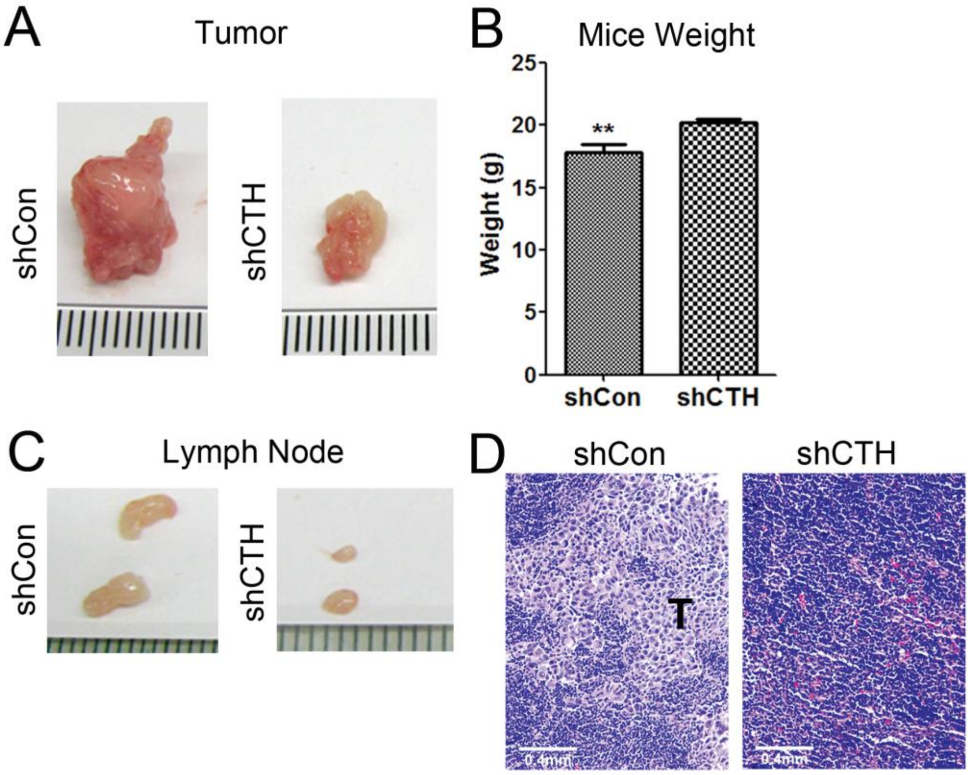

Appendix Fig S3. Prostate orthotopic implantation of PC3 cells with reduced CTH expression decreases tumor growth and distant metastasis. $1x10^6$ PC3 cells with control or CTH knockdown were orthotopically injected into mouse prostate for 60 days. (A) Images of PC3 xenografts (shCon or shCTH) (B) The mice weight were compared. Data are presented as means ± SEM (n = 10 mice per group). Student's t-test was used for the statistical analysis (**P<0.01). (C) Images of paraaortic lymph nodes of mice orthotopically implanted with PC3-shCon or PC3-shCTH cells. (D) Paraaortic lymph nodes of nude mice orthotopically implanted with PC3-shCon (left panel) or PC3-shCTH cells (right panel). Images show H&E staining of paraaortic lymph node sections. T: metastatic tumor cells (scale bar: 0.4mm).

Appendix Fig S4

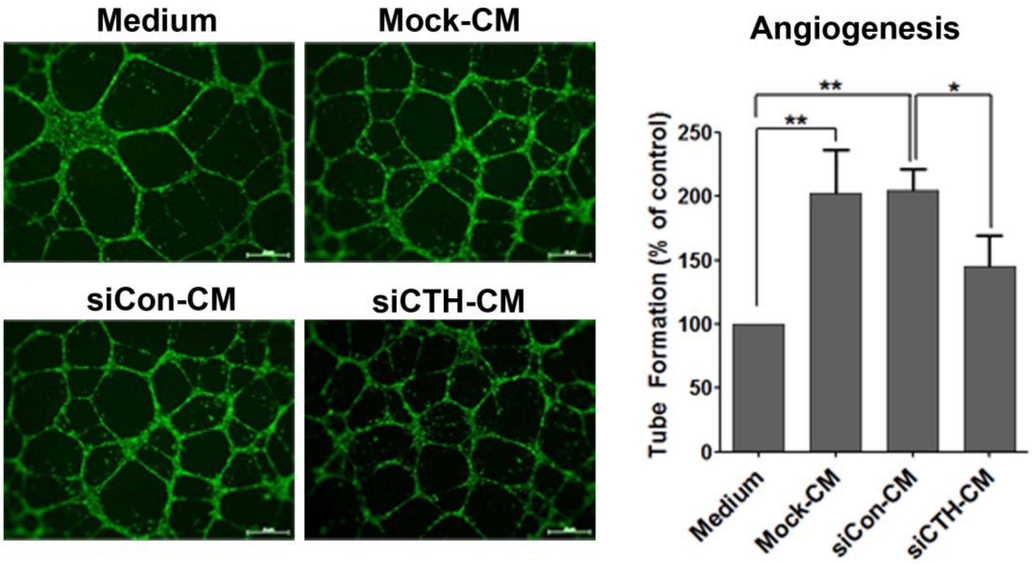

Appendix Fig S4. Tube formation assay by HUVEC cells co-culturing in conditional medium from PC3-B2 cells with CTH knockdown. HUVECs seeded on Matrigel and incubated with PC3-B2 derived conditional medium. Cells were photographed 16 hours after seeding, and the number of nodes formed was counted and averaged. Data shown represent the means ± SD (n=3 biological replicates). ANOVA followed by Tukey's post-hoc test was used for the statistical analysis. (*P<0.05; **P<0.01). Representative photos are shown on the left panel.

Real-Time RT-PCR primer sequences (SYBR)		
	Forward	Reverse
IL1β	CTCTTCAGCCAATCTTCATT	CCACTGTAATAAGCCATCATT
VEGF	CTGCTCTACCTCCACCAT	ATGAACTTCACCACTTCGT
MMP13	AAGGAGCATGGCGACTTCTA	GGTCCTTGGAGTGGTCAAGA
CTH	GAAGACCTACTGGAAGAT	GGAATACTAGCTGTGACT
RPS3	CTGGAGTTGAGGTGCGAGTT	GGCCCTTCTCACCAAGAACA
actin	CGGCATCGTCACCAACTG	TCTCAAACATGATCTGGGTCATCT

Cloning primer sequences	
PCDNA-CTH-F	GCTAGAATTCCTTTCGCGGTTCAGC
PCDNA-CTH-R	ATATCTCGAGCTAGCTGTGACTTCCACTTG
PCDNA-p65-F	ATATAAGCTTCATGGACGAACTGTTCCCC
PCDNA-p65-R	ATATCTCGAGTTAGGAGCTGATCTGACTC

Mutagenesis primer sequences	
p65-C38S-F	TTCCGCTACAAGTCCGAGGG
p65-C38S-R	AAGGCGATGTTCAGGCACCC
p65-sgRNAresist-F	GGTGCAGAAAGAGGATATCGAGGTGTATTTCACG
p65-sgRNAresist-R	CGTGAAATACACCTCGATATCCTCTTTCTGCACC
CTH-Q240E-F	CGTTTCTTGGAAAACTCTCTT
CTH-Q240E-R	AAGAGAGTTTTCCAAGAAACG

siRNA sequence	
CTH-1	GGAGCUGAUAUUUCUAUGU
CTH-2	CCUGGUGUCUGUUAAUUGU
RPS3	GCAUCAAGGUGAAGAUCAU

sgRNA sequence	
p65 sgRNA	GCTTGCGAAAAGGAGCCTCGGG

pRFP-C-RS-shCTH sequence	
CTH	GGCTTAGAGGATGAGGAAGACCTACTGGA

Appendix Table S1. Primers, siRNAs, and sgRNA used in this study.

Patient No.	CTH (T)	gender (T)	vital_status (T)	last_contact_days_to (T)
1	254.3021	MALE	Alive	1
2	346.211	MALE	Alive	1
3	264.2643	MALE	Alive	9
4	244.6081	MALE	Alive	22
5	160.7762	MALE	Alive	23
6	296.3728	MALE	Alive	24
7	100.5314	MALE	Alive	27
8	428.2528	MALE	Alive	28
9	174.2976	MALE	Alive	29
10	131.3192	MALE	Alive	29
11	107.448	MALE	Alive	31
12	179.2786	MALE	Alive	31
13	225.8082	MALE	Alive	31
14	268.1401	MALE	Alive	31
15	312.7435	MALE	Alive	32
16	118.8392	MALE	Alive	32
17	187.3434	MALE	Alive	34
18	82.6625	MALE	Alive	34
19	174.8289	MALE	Alive	34
20	121.1765	MALE	Alive	35
21	91.2017	MALE	Alive	38
22	185.6869	MALE	Alive	41
23	204.4135	MALE	Alive	41
24	205.5866	MALE	Alive	42
25	199.9834	MALE	Alive	44
26	184.9372	MALE	Alive	44
27	204.2904	MALE	Alive	45
28	82.5726	MALE	Alive	46
29	165.1201	MALE	Alive	48
30	133.8726	MALE	Alive	49
31	198.1154	MALE	Alive	50
32	236.892	MALE	Alive	51
33	202.3627	MALE	Alive	51
34	284.4805	MALE	Alive	52
35	142.4382	MALE	Alive	52

36	124.1554	MALE	Alive	53
37	109.992	MALE	Alive	54
38	264.1869	MALE	Alive	54
39	104.707	MALE	Alive	54
40	183.7183	MALE	Alive	56
41	209.5041	MALE	Alive	57
42	213.7712	MALE	Alive	60
43	127.8317	MALE	Alive	60
44	97.5783	MALE	Alive	60
45	112.3376	MALE	Alive	61
46	70.5538	MALE	Alive	62
47	192.4306	MALE	Alive	62
48	165.3055	MALE	Alive	62
49	160.0982	MALE	Alive	62
50	193.6124	MALE	Alive	62
51	131.1842	MALE	Alive	63
52	214.9357	MALE	Alive	63
53	179.8616	MALE	Alive	64
54	188.7694	MALE	Alive	66
55	89.3973	MALE	Alive	67
56	125	MALE	Alive	68
57	109.1196	MALE	Alive	70
58	98.0575	MALE	Alive	70
59	238.9468	MALE	Alive	70
60	96.3391	MALE	Alive	71
61	144.6261	MALE	Alive	72
62	204.8742	MALE	Alive	76
63	184.9926	MALE	Alive	77
64	236.2149	MALE	Alive	79
65	291.5746	MALE	Alive	81
66	197.7492	MALE	Alive	82
67	257.5198	MALE	Alive	83
68	129.1161	MALE	Alive	86
69	202.1637	MALE	Alive	89
70	283.6308	MALE	Alive	91
71	234.3626	MALE	Alive	91
72	125.9164	MALE	Alive	91

73	124.6471	MALE	Alive	91
74	162.5222	MALE	Alive	91
75	376.6441	MALE	Alive	91
76	219.5652	MALE	Alive	93
77	228.1414	MALE	Alive	95
78	70.0062	MALE	Alive	96
79	81.3008	MALE	Alive	97
80	167.7439	MALE	Alive	98
81	224.1435	MALE	Alive	101
82	201.2443	MALE	Alive	102
83	75.488	MALE	Alive	104
84	211.9018	MALE	Alive	106
85	136.3997	MALE	Alive	106
86	156.0491	MALE	Alive	110
87	178.7611	MALE	Alive	112
88	125.605	MALE	Alive	112
89	146.4336	MALE	Alive	112
90	199.6248	MALE	Alive	113
91	231.4669	MALE	Alive	114
92	109.0556	MALE	Alive	115
93	133.3383	MALE	Alive	117
94	183.4962	MALE	Alive	119
95	133.4263	MALE	Alive	121
96	145.2213	MALE	Alive	122
97	161.7397	MALE	Alive	123
98	78.2504	MALE	Alive	126
99	144.3159	MALE	Alive	128
100	187.6457	MALE	Alive	129
101	31.2734	MALE	Alive	131
102	256.861	MALE	Alive	132
103	149.5465	MALE	Alive	132
104	134.2505	MALE	Alive	134
105	65.4321	MALE	Alive	134
106	195.6615	MALE	Alive	145
107	158.2734	MALE	Alive	148
108	246.4247	MALE	Alive	149
109	162.2696	MALE	Alive	150

110	180.9211	MALE	Alive	150
111	225.9578	MALE	Alive	156
112	162.5644	MALE	Alive	158
113	154.8747	MALE	Alive	162
114	123.4136	MALE	Alive	162
115	170.1733	MALE	Alive	167
116	99.9653	MALE	Alive	167
117	170.3171	MALE	Alive	169
118	126.4098	MALE	Alive	170
119	127.9903	MALE	Alive	170
120	168.2809	MALE	Alive	172
121	166.6285	MALE	Alive	172
122	142.4837	MALE	Alive	173
123	221.9186	MALE	Alive	175
124	184.2155	MALE	Alive	176
125	114.8126	MALE	Alive	177
126	77.8286	MALE	Alive	178
127	232.9076	MALE	Alive	182
128	234.0922	MALE	Alive	183
129	158.9546	MALE	Alive	183
130	133.0449	MALE	Alive	187
131	158.7105	MALE	Alive	187
132	174.2059	MALE	Alive	191
133	110.07	MALE	Alive	196
134	216.4024	MALE	Alive	196
135	196.2474	MALE	Alive	197
136	188.4647	MALE	Alive	202
137	187.6862	MALE	Alive	203
138	230.3945	MALE	Alive	204
139	166.442	MALE	Alive	204
140	184.3736	MALE	Alive	206
141	191.248	MALE	Alive	211
142	215.7429	MALE	Alive	212
143	206.9623	MALE	Alive	213
144	92.1848	MALE	Alive	213
145	235.8452	MALE	Alive	216
146	198.4474	MALE	Alive	216

147	161.1857	MALE	Alive	217
148	46.1341	MALE	Alive	223
149	152.691	MALE	Alive	229
150	74.7619	MALE	Alive	229
151	150.4124	MALE	Alive	230
152	258.1602	MALE	Alive	233
153	89.3742	MALE	Alive	244
154	166.5988	MALE	Alive	245
155	221.3302	MALE	Alive	249
156	118.2409	MALE	Alive	256
157	195.9478	MALE	Alive	259
158	67.7849	MALE	Alive	261
159	108.1844	MALE	Alive	263
160	179.825	MALE	Alive	264
161	77.5862	MALE	Alive	269
162	116.1731	MALE	Alive	270
163	330.0377	MALE	Alive	273
164	177.1859	MALE	Alive	278
165	126.5732	MALE	Alive	279
166	188.7415	MALE	Alive	279
167	200.307	MALE	Alive	285
168	238.6844	MALE	Alive	285
169	132.5148	MALE	Alive	287
170	171.9653	MALE	Alive	289
171	109.529	MALE	Alive	290
172	148.8398	MALE	Alive	295
173	200.4853	MALE	Alive	295
174	115.7663	MALE	Alive	295
175	147.9132	MALE	Alive	295
176	62.8364	MALE	Alive	299
177	149.4208	MALE	Alive	302
178	162.5509	MALE	Alive	304
179	157.767	MALE	Alive	308
180	189.5593	MALE	Alive	310
181	227.5892	MALE	Alive	314
182	194.7566	MALE	Alive	316
183	165.3272	MALE	Alive	321

184	82.766	MALE	Alive	324
185	328.1899	MALE	Alive	350
186	59.7661	MALE	Alive	350
187	97.057	MALE	Alive	352
188	192.4623	MALE	Alive	353
189	169.1437	MALE	Alive	356
190	181.4103	MALE	Alive	356
191	171.1027	MALE	Alive	358
192	158.4706	MALE	Alive	365
193	260.2802	MALE	Alive	371
194	51.6579	MALE	Alive	386
195	169.3792	MALE	Alive	390
196	96.1721	MALE	Alive	393
197	90.7372	MALE	Alive	394
198	210.9323	MALE	Alive	395
199	258.1674	MALE	Alive	396
200	146.1325	MALE	Alive	396
201	163.6967	MALE	Alive	397
202	109.8655	MALE	Alive	405
203	228.1298	MALE	Alive	405
204	104.2506	MALE	Alive	405
205	236.7455	MALE	Alive	412
206	121.0124	MALE	Alive	417
207	130.6598	MALE	Alive	422
208	105.2813	MALE	Alive	425
209	56.0803	MALE	Alive	427
210	84.0841	MALE	Alive	428
211	145.5236	MALE	Alive	437
212	210.5342	MALE	Alive	448
213	253.9286	MALE	Alive	448
214	171.8728	MALE	Alive	452
215	95.0081	MALE	Alive	454
216	177.3227	MALE	Alive	457
217	254.8726	MALE	Alive	458
218	131.8066	MALE	Alive	459
219	90.6103	MALE	Alive	463
220	231.4022	MALE	Alive	474

221	239.7396	MALE	Alive	476
222	48.5372	MALE	Alive	476
223	176.572	MALE	Alive	481
224	134.9907	MALE	Alive	483
225	264.4628	MALE	Alive	485
226	260.0791	MALE	Alive	486
227	100.7943	MALE	Alive	487
228	98.6409	MALE	Alive	488
229	97.3947	MALE	Alive	491
230	26.455	MALE	Alive	503
231	184.1764	MALE	Alive	505
232	195.1804	MALE	Alive	508
233	153.8741	MALE	Alive	510
234	122.0845	MALE	Alive	512
235	139.8573	MALE	Alive	512
236	173.0449	MALE	Alive	518
237	125.5622	MALE	Alive	518
238	196.7825	MALE	Alive	519
239	118.8707	MALE	Alive	523
240	142.288	MALE	Alive	524
241	166.1775	MALE	Alive	526
242	143.932	MALE	Alive	526
243	159.5745	MALE	Alive	528
244	198.8124	MALE	Alive	530
245	171.4643	MALE	Alive	533
246	178.6618	MALE	Alive	533
247	77.0877	MALE	Alive	536
248	196.6774	MALE	Alive	537
249	212.968	MALE	Alive	542
250	84.7657	MALE	Alive	549
251	166.0526	MALE	Alive	554
252	107.139	MALE	Alive	560
253	214.8303	MALE	Alive	567
254	49.7716	MALE	Alive	570
255	198.4405	MALE	Alive	589
256	238.0268	MALE	Alive	595
257	244.4334	MALE	Alive	598

258	30.4248	MALE	Alive	602
259	148.7965	MALE	Alive	606
260	183.6926	MALE	Alive	614
261	154.0415	MALE	Alive	615
262	342.9454	MALE	Alive	621
263	68.3024	MALE	Alive	624
264	125	MALE	Alive	636
265	128.1573	MALE	Alive	637
266	193.6275	MALE	Alive	643
267	168.5393	MALE	Alive	655
268	149.1491	MALE	Alive	662
269	208.482	MALE	Alive	663
270	234.1311	MALE	Alive	663
271	162.4185	MALE	Alive	664
272	177.2632	MALE	Alive	664
273	191.3818	MALE	Alive	665
274	185.7143	MALE	Alive	668
275	140.3107	MALE	Alive	668
276	209.8569	MALE	Alive	671
277	138.8399	MALE	Alive	671
278	219.8834	MALE	Alive	674
279	96.8222	MALE	Alive	686
280	192.623	MALE	Alive	692
281	97.2654	MALE	Alive	692
282	117.5337	MALE	Alive	693
283	302.3093	MALE	Alive	700
284	69.8599	MALE	Alive	709
285	118.2864	MALE	Alive	717
286	96.3145	MALE	Alive	721
287	204.6465	MALE	Alive	724
288	173.0808	MALE	Alive	724
289	208.6173	MALE	Alive	729
290	225.1472	MALE	Alive	731
291	212.2025	MALE	Alive	731
292	131.6407	MALE	Alive	738
293	113.1145	MALE	Alive	740
294	109.4359	MALE	Alive	756

295	145.2442	MALE	Alive	757
296	198.9531	MALE	Alive	762
297	84.2154	MALE	Alive	770
298	241.2595	MALE	Alive	771
299	145.9016	MALE	Alive	774
300	258.3371	MALE	Alive	775
301	157.5071	MALE	Alive	782
302	234.217	MALE	Alive	783
303	260.0685	MALE	Alive	788
304	406.588	MALE	Alive	790
305	84.118	MALE	Alive	790
306	127.3366	MALE	Alive	798
307	159.0816	MALE	Alive	804
308	173.6876	MALE	Alive	804
309	202.5547	MALE	Alive	805
310	143.5644	MALE	Alive	806
311	142.993	MALE	Alive	821
312	339.8268	MALE	Alive	821
313	135.718	MALE	Alive	822
314	163.5323	MALE	Alive	822
315	193.144	MALE	Alive	822
316	333.9401	MALE	Alive	824
317	141.2893	MALE	Alive	834
318	84.2427	MALE	Alive	840
319	209.1272	MALE	Alive	841
320	119.6411	MALE	Alive	846
321	130.6865	MALE	Alive	846
322	216.9395	MALE	Alive	850
323	126.6365	MALE	Alive	851
324	160.4301	MALE	Alive	854
325	150.2003	MALE	Alive	857
326	264.1068	MALE	Alive	868
327	144.4405	MALE	Alive	875
328	174.2892	MALE	Alive	882
329	138.427	MALE	Alive	889
330	81.0664	MALE	Alive	913
331	206.4705	MALE	Alive	917

332	197.6927	MALE	Alive	922
333	115.9651	MALE	Alive	923
334	207.3245	MALE	Alive	926
335	193.6049	MALE	Alive	930
336	106.8085	MALE	Alive	934
337	146.0348	MALE	Alive	943
338	137.3414	MALE	Alive	958
339	181.8497	MALE	Alive	972
340	165.874	MALE	Alive	972
341	194.7515	MALE	Alive	974
342	145.7555	MALE	Alive	986
343	173.6896	MALE	Alive	993
344	178.6765	MALE	Alive	995
345	97.3016	MALE	Alive	999
346	106.2048	MALE	Alive	1000
347	206.3348	MALE	Alive	1001
348	187.4911	MALE	Alive	1004
349	141.2982	MALE	Alive	1008
350	195.4023	MALE	Alive	1014
351	147.2031	MALE	Alive	1019
352	222.6981	MALE	Alive	1033
353	162.0016	MALE	Alive	1049
354	55.2036	MALE	Alive	1054
355	229.3982	MALE	Alive	1061
356	152.1291	MALE	Alive	1063
357	250.2596	MALE	Alive	1065
358	171.4774	MALE	Alive	1102
359	215.502	MALE	Alive	1108
360	337.0056	MALE	Alive	1109
361	137.1974	MALE	Alive	1117
362	207.2109	MALE	Alive	1124
363	158.4343	MALE	Alive	1126
364	115.7294	MALE	Alive	1131
365	126.4874	MALE	Alive	1147
366	115.7025	MALE	Alive	1153
367	90.9879	MALE	Alive	1155
368	159.6391	MALE	Alive	1157

369	153.618	MALE	Alive	1164
370	231.7687	MALE	Alive	1173
371	224.6466	MALE	Alive	1184
372	266.3062	MALE	Alive	1195
373	196.3282	MALE	Alive	1206
374	176.3433	MALE	Alive	1215
375	173.8038	MALE	Alive	1230
376	250.838	MALE	Alive	1237
377	55.8227	MALE	Alive	1247
378	177.4682	MALE	Alive	1253
379	144.9838	MALE	Alive	1254
380	347.7619	MALE	Alive	1259
381	159.2357	MALE	Alive	1261
382	168.5363	MALE	Alive	1272
383	174.2138	MALE	Alive	1280
384	177.3952	MALE	Alive	1296
385	118.8487	MALE	Alive	1328
386	140.8998	MALE	Alive	1332
387	188.5559	MALE	Alive	1338
388	154.3917	MALE	Alive	1339
389	131.654	MALE	Alive	1349
390	98.298	MALE	Alive	1359
391	179.4654	MALE	Alive	1373
392	234.375	MALE	Alive	1375
393	192.1732	MALE	Alive	1384
394	187.6462	MALE	Alive	1386
395	64.2471	MALE	Alive	1393
396	134.6824	MALE	Alive	1395
397	135.738	MALE	Alive	1399
398	164.559	MALE	Alive	1438
399	154.5548	MALE	Alive	1441
400	190.8084	MALE	Alive	1442
401	106.1125	MALE	Alive	1461
402	237.5829	MALE	Alive	1491
403	162.7998	MALE	Alive	1491
404	177.6225	MALE	Alive	1498
405	170.0796	MALE	Alive	1501

406	194.1568	MALE	Alive	1518
407	180.2735	MALE	Alive	1559
408	168.8022	MALE	Alive	1563
409	93.6849	MALE	Alive	1572
410	178.3354	MALE	Alive	1608
411	109.2991	MALE	Alive	1611
412	142.9655	MALE	Alive	1621
413	223.7106	MALE	Alive	1635
414	98.6141	MALE	Alive	1649
415	108.7585	MALE	Alive	1659
416	220.5641	MALE	Alive	1669
417	212.9838	MALE	Alive	1690
418	48.4373	MALE	Alive	1718
419	212.2736	MALE	Alive	1770
420	79.7353	MALE	Alive	1776
421	64.8314	MALE	Alive	1776
422	240.2288	MALE	Alive	1815
423	233.2083	MALE	Alive	1842
424	186.3231	MALE	Alive	1853
425	149.022	MALE	Alive	1860
426	56.7787	MALE	Alive	1879
427	209.8248	MALE	Alive	1890
428	107.0703	MALE	Alive	1891
429	147.7585	MALE	Alive	1897
430	111.4447	MALE	Alive	1925
431	146.7033	MALE	Alive	1943
432	139.9257	MALE	Alive	1946
433	85.6134	MALE	Alive	1949
434	123.6897	MALE	Alive	1961
435	283.3798	MALE	Alive	1973
436	187.8006	MALE	Alive	2037
437	51.4113	MALE	Alive	2040
438	177.3103	MALE	Alive	2051
439	72.9589	MALE	Alive	2056
440	46.5208	MALE	Alive	2068
441	174.7652	MALE	Alive	2110
442	194.2446	MALE	Alive	2170

443	127.9676	MALE	Alive	2183
444	118.2266	MALE	Alive	2211
445	165.5481	MALE	Alive	2309
446	131.4128	MALE	Alive	2315
447	163.7401	MALE	Alive	2328
448	297.6721	MALE	Alive	2364
449	136.0025	MALE	Alive	2425
450	269.0698	MALE	Alive	2542
451	139.5724	MALE	Alive	2553
452	167.5353	MALE	Alive	2576
453	148.314	MALE	Alive	2620
454	128.0851	MALE	Alive	2704
455	74.8384	MALE	Alive	2753
456	183.3741	MALE	Alive	2859
457	148.2667	MALE	Alive	3247
458	157.1364	MALE	Alive	3447
459	100.0547	MALE	Alive	3487
460	196.5959	MALE	Alive	3524
461	142.7429	MALE	Alive	4604
462	187.0794	MALE	Dead	628
463	153.4058	MALE	Dead	728
464	289.8361	MALE	Dead	791
465	183.1073	MALE	Dead	1329
466	186.3643	MALE	Dead	1855
467	172.3044	MALE	Dead	875
468	195.0005	MALE	Dead	146
469	151.8738	MALE	Dead	3467

Appendix Table S2. Clinical data for 469 prostate cancer cases.
