# Supplementary material for: Dysregulation of cystathionine γ‐lyase promotes prostate cancer progression and metastasis
Source: EMBO Rep. 2019 Aug 29;20(10):e45986. doi: 10.15252/embr.201845986 (PMC6776913; doi:10.15252/embr.201845986)
Supplement: Supplementary file 2 — Expanded View Figures PDF [file EMBR-20-e45986-s002.pdf]

Expanded View Figures

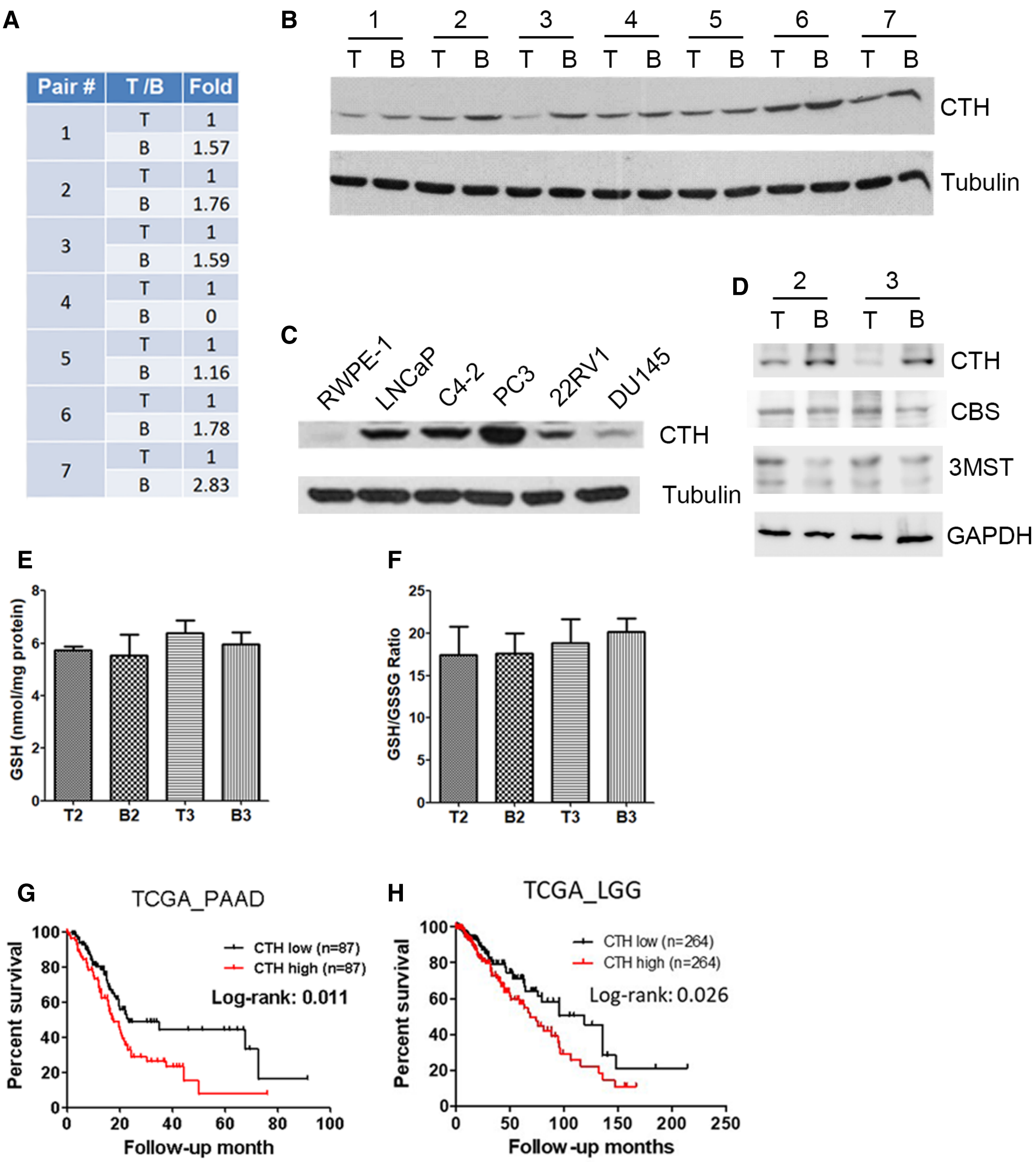

Figure EV1.

 © 2019 The Authors

Figure EV1. CTH level is elevated in metastatic prostate cancer cells and correlates with poor survival in other cancer types.

A Microarray analysis showing CTH mRNA expression in seven pairs of PC3 cells grown in bone marrow (B line), compared to cells grown in the orthotopic implanted prostate tumor (T line). The fold changes indicated each T line to B line of CTH expression.

B Western blot analysis of CTH expression in seven pairs of PC3-B lines and PC3-T lines.

C Western blot analysis of CTH expression in prostate normal epithelial cells, RWPE-1, together with five prostate cancer cells, including LNCaP, C4-2, PC3, 22RV1, and DU145 cells.

D Western blot analysis of CTH, CBS, and 3MST expression in PC3-T2, PC3-B2, PC3-T3, and PC3-B3 lines.

E, F Total glutathione (GSH) content and the ratio of reduced glutathione to oxidized glutathione (GSH/GSSG). Cells were seeded at a density of 5×10^3 cells per 96-well plate overnight; then, cells were lysed to measure the GSH and GSSG levels. Data indicate total glutathione concentration normalized to total protein content (E) and the ratio of GSH to GSSG normalized to total protein content (F). Data shown represent the normalized means ± SD (n = 3 biological replicates).

G, H Association of CTH expression with poor survival in pancreatic adenocarcinoma (PAAD) and lower grade glioma (LGG). Kaplan–Meier survival analysis of PAAD (G) and LGG (H) patients with high or low CTH expression. The RNA-seq data of CTH were downloaded from the TCGA database as described in the section of Materials and Methods. The statistical significance was determined using the chi-square test.

Source data are available online for this figure.

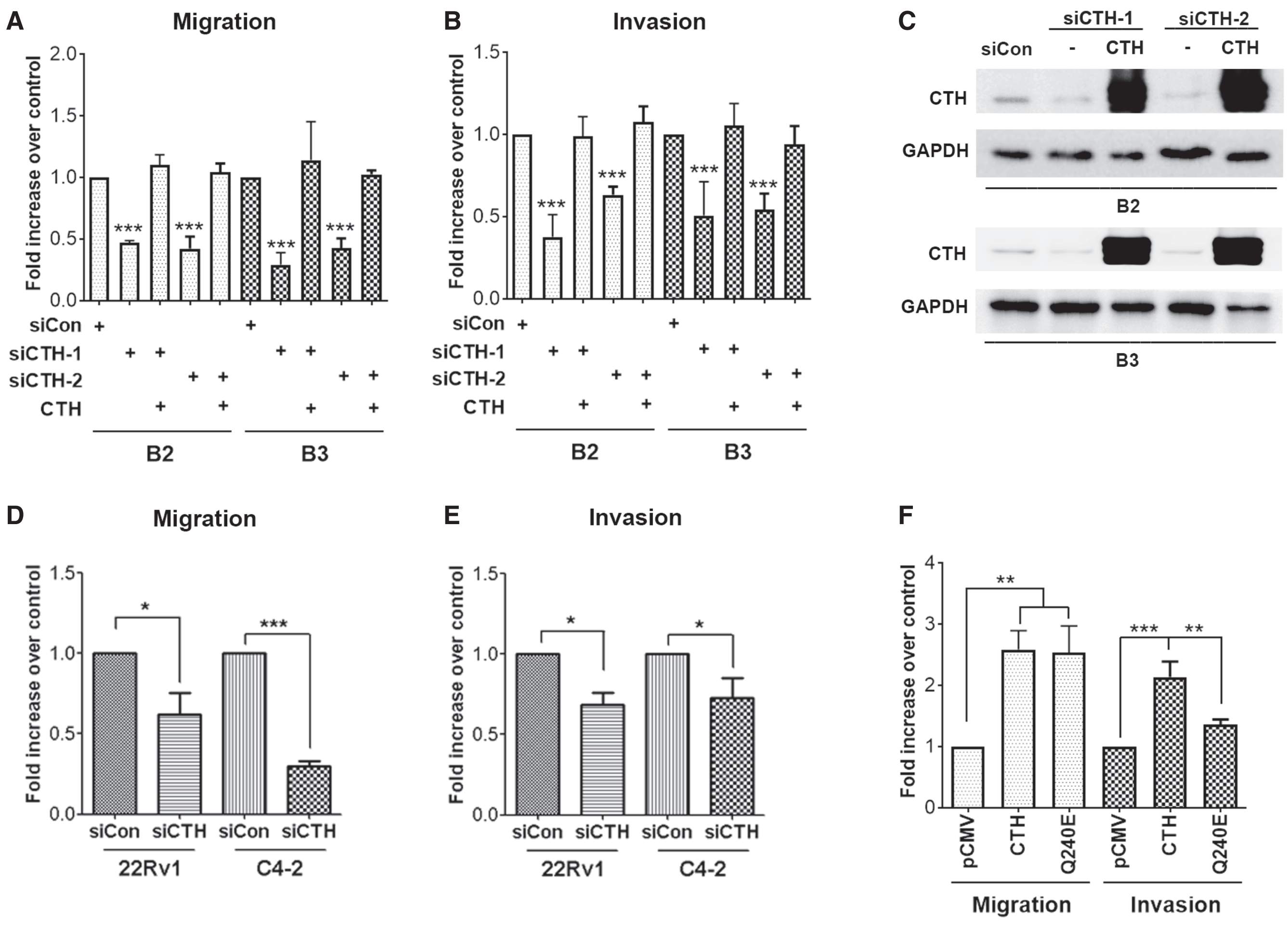

Figure EV2. CTH expression stimulates cell migration and invasion in prostate cancer cells.

A, B PC3-B2 and PC3-B3 cells were transfected with control siRNA, siCTH-1, 2, or pCMV-CTH-HA, and then incubated for migration (A) and invasion assay (B). Data shown represent the normalized means ± SD (n = 3 biological replicates). ANOVA followed by Tukey's post hoc test was used (***$P < 0.001$).

C Western blot analysis of the CTH levels in PC3-B2 and PC3-B3 cells transfected with control siRNA, siCTH-1, 2, or pCMV-CTH-HA.

D, E 22Rv1 and C4-2 cells were transfected with control or siCTH-1 and then were subjected to migration (D) and invasion (E) assays. Data shown represent the means ± SD (n = 3 biological replicates). Student's t-test was used for the statistical analysis (*$P < 0.05$; ***$P < 0.001$).

F PC3 cells were transfected with HA-CTH, HA-CTH^{Q240E}, or pCMV-HA vector control and then incubated for migration and invasion assay. Data shown represent the means ± SD (n = 3 biological replicates). ANOVA followed by Tukey's post hoc test was used (**$P < 0.01$; ***$P < 0.001$).

Source data are available online for this figure.

© 2019 The Authors

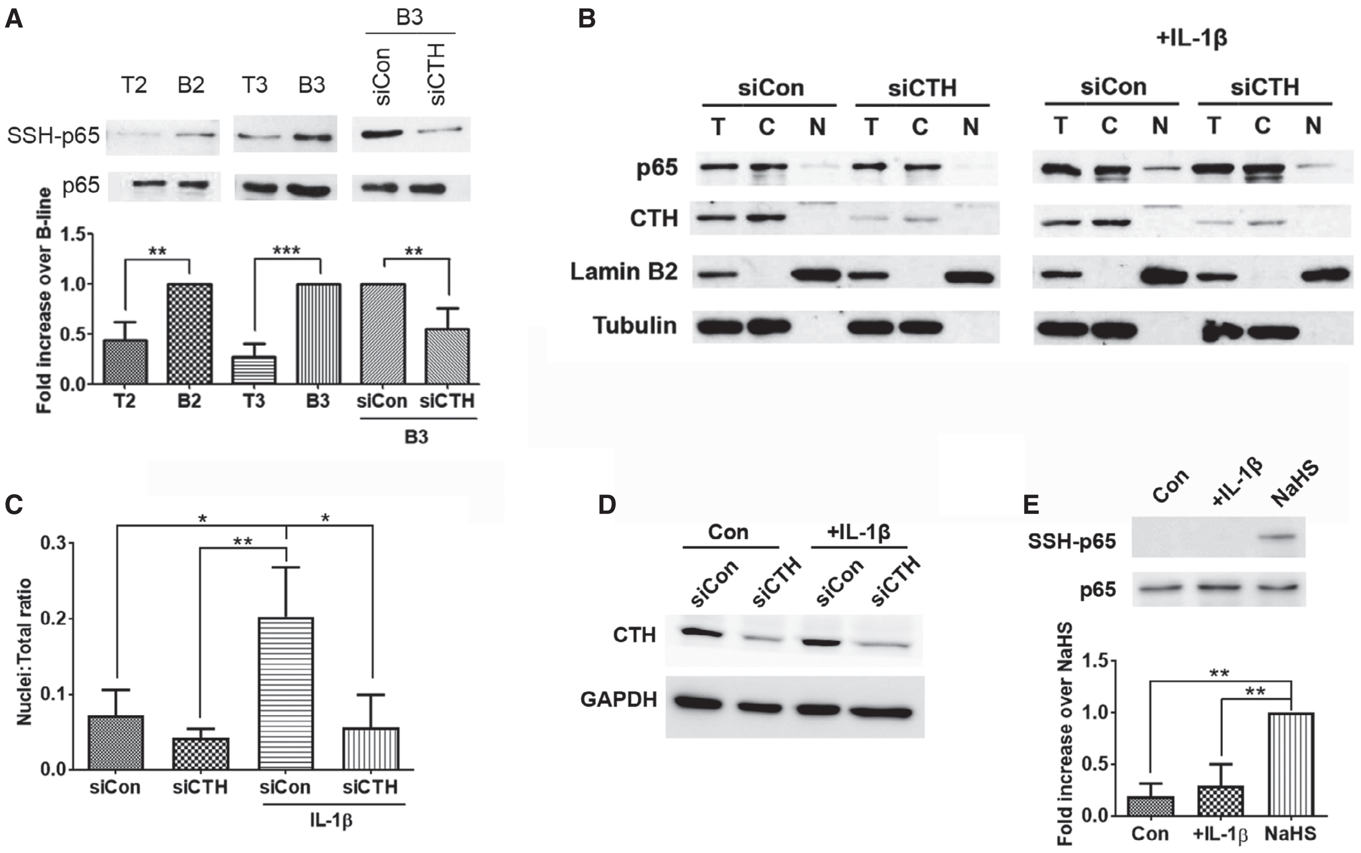

Figure EV3. Knockdown of CTH decreases IL-1β-stimulated p65 nuclear translocation.

A Upper: PC3-T2, PC3-B2, PC3-T3, PC3-B3 cells or PC3-B3 cells with CTH or control siRNA transfection were subjected to the modified biotin switch assay with the antibody against p65 to detect S-sulfhydration. Bottom: Quantitative analysis of SSH-p65 protein level, and normalized with total p65 level. Histograms represent normalized means ± SD (n = 3 biological replicates). Student's t-test was used for the statistical analysis (**$P < 0.01$; ***$P < 0.001$).

B PC3 cells were treated with 20 ng/ml IL-1β for 1 h after 48 h post-transfection. Cells were then subject to fractionation, and the extracts from the nuclear and cytoplasmic compartments were analyzed by Western blotting using the indicated antibodies. Lamin B2 and tubulin serve as the marker of the nuclear and cytoplasmic compartments, respectively. T: total, N: nuclear fraction, and C: cytoplasmic fraction.

C Quantitative analysis of the cellular fractionation results from (B). The p65 nuclear protein level was normalized with p65 level in the total cell lysate. Histograms represent means ± SD (n = 3 biological replicates). ANOVA followed by Tukey's post hoc test was used for the statistical analysis (*$P < 0.05$; **$P < 0.01$).

D The expression of CTH upon IL-1β stimulation. PC3 cells were treated with 20 ng/ml IL-1β for 24 h at 48 h post-transfection with siCTH. Cell lysates were then analyzed by Western blotting using an antibody against CTH.

E Upper: PC3 cells treated with 20 ng/ml IL-1β for 24 h were subjected to the modified biotin switch assay with the antibody against p65 to detect S-sulfhydration. PC3 cells treated with 1 μM NaHS for 1 h were used as positive control. Bottom: Histograms represent normalized means ± SD (n = 3 biological replicates). ANOVA followed by Tukey's post hoc test was used for the statistical analysis (**$P < 0.01$).

Source data are available online for this figure.

 © 2019 The Authors

Figure EV4. Treatment of H_2S induces PC3 cell invasion through NF-κB-mediated signaling pathways.

A 22Rv1 and C4-2 cells were incubated for invasion assay for 16–18 h. DMEM/10% FBS, together with 1 μM NaHS served as a chemoattractant.
B PC3 cells were incubated for invasion assay for 24 h. DMEM/10% FBS, together with 1 or 10 μM GYY4137, served as a chemoattractant.
C Top: PC3 cells treated with 1 nM–1 mM NaHS for 1 h were subjected to the modified biotin switch assay with the antibody against p65 to detect S-sulfhydration. Bottom: Quantitative analysis of SSH-p65 protein level, and normalized with total p65 level.
D PC3 cells were treated with 1 μM NaHS for 24 h in the absence of serum. Subcellular localization of p65 was detected by immunocytochemistry. Nuclei were counterstained with DAPI. Scale bar: 25 μm.
E Nuclear translocation of p65 was scored by counting the number of nuclear positive stained cells of p65 to the total number of cells in random microscopic fields.
F PC3 cells were incubated for invasion assay for 24 h. DMEM/10% FBS, together with 1 μM GYY4137 and NF-κB inhibitors (50 μg/ml SN50, or 100 nM QNZ), served as a chemoattractant.
G Real-time RT–PCR analysis for IL-1β, MMP-13, VEGF, and RPS3 mRNA level in PC3 cells with control or RPS3 knockdown.
H Left: PC3 cells with p65 knockout with p65 C38S mutant and PC3 parental cells were treated with 100 μM NaHS for 1 h and subjected to the modified biotin switch assay with the antibody against p65 to detect S-sulfhydration. Right: Quantitative analysis of SSH-p65 protein level, and normalized with total p65 level.
I PC3 cells with p65 knockout were transfected with vector, p65 wild-type, or p65 C38S mutant and then exposed to IL-1β (20 ng/ml) for 1 h. Subcellular localization of p65 was detected by immunocytochemistry. Nuclei were counterstained with DAPI. The representative images are shown. Scale bars: 10 μm.
J Nuclear translocation of p65 was scored by counting the nuclear positive stained cells and the total number of cells quantified in random microscopic fields.

Data information: (A–C, E–H, J) Data shown represent the means ± SD (n = 3 biological replicates). Student's t-test (A, E, G) or ANOVA followed by Tukey's post hoc test (B, F, H, I) was used for statistical analysis (*$P < 0.05$; **$P < 0.01$; ***$P < 0.001$).
Source data are available online for this figure.

© 2019 The Authors

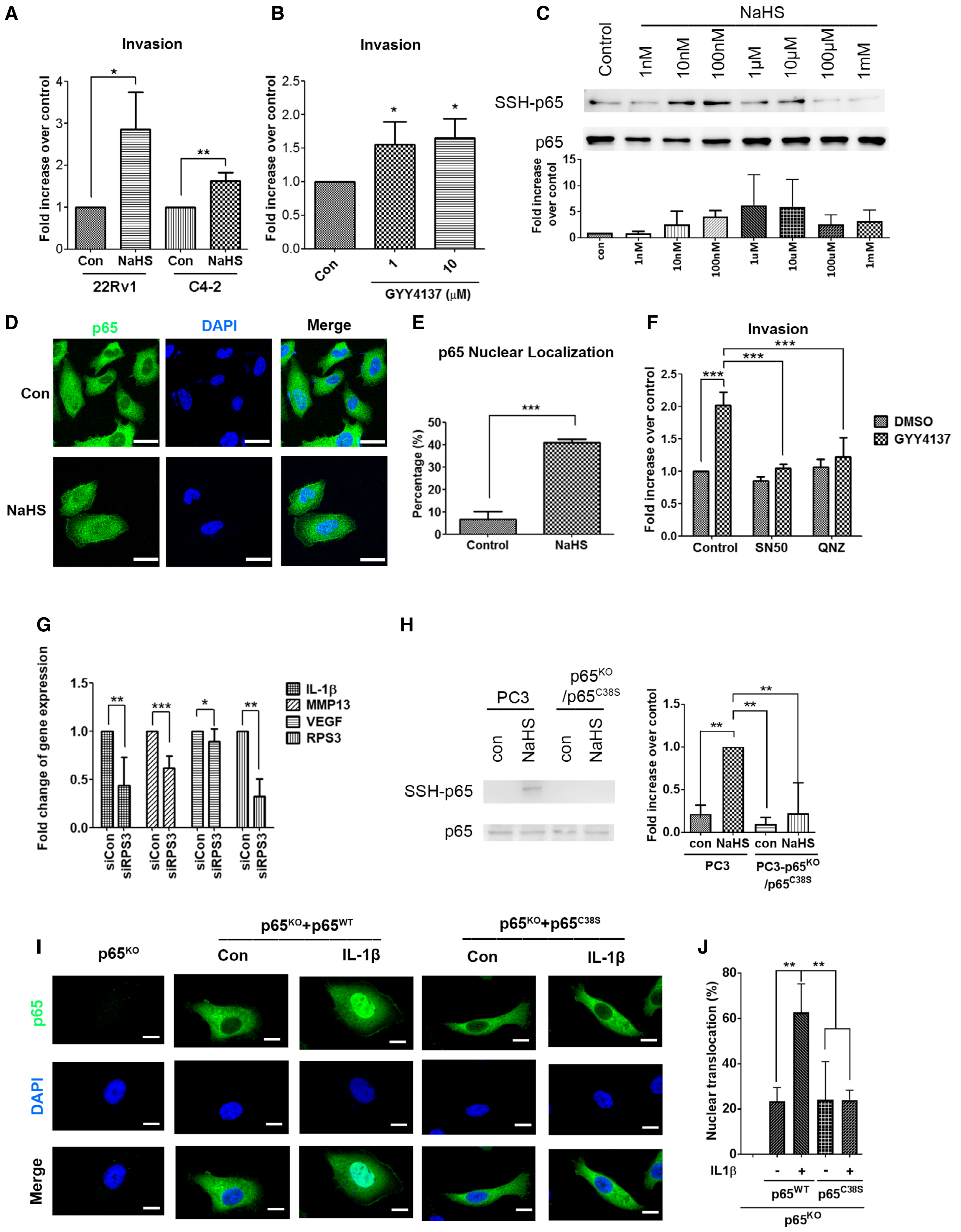

Figure EV4.

 © 2019 The Authors

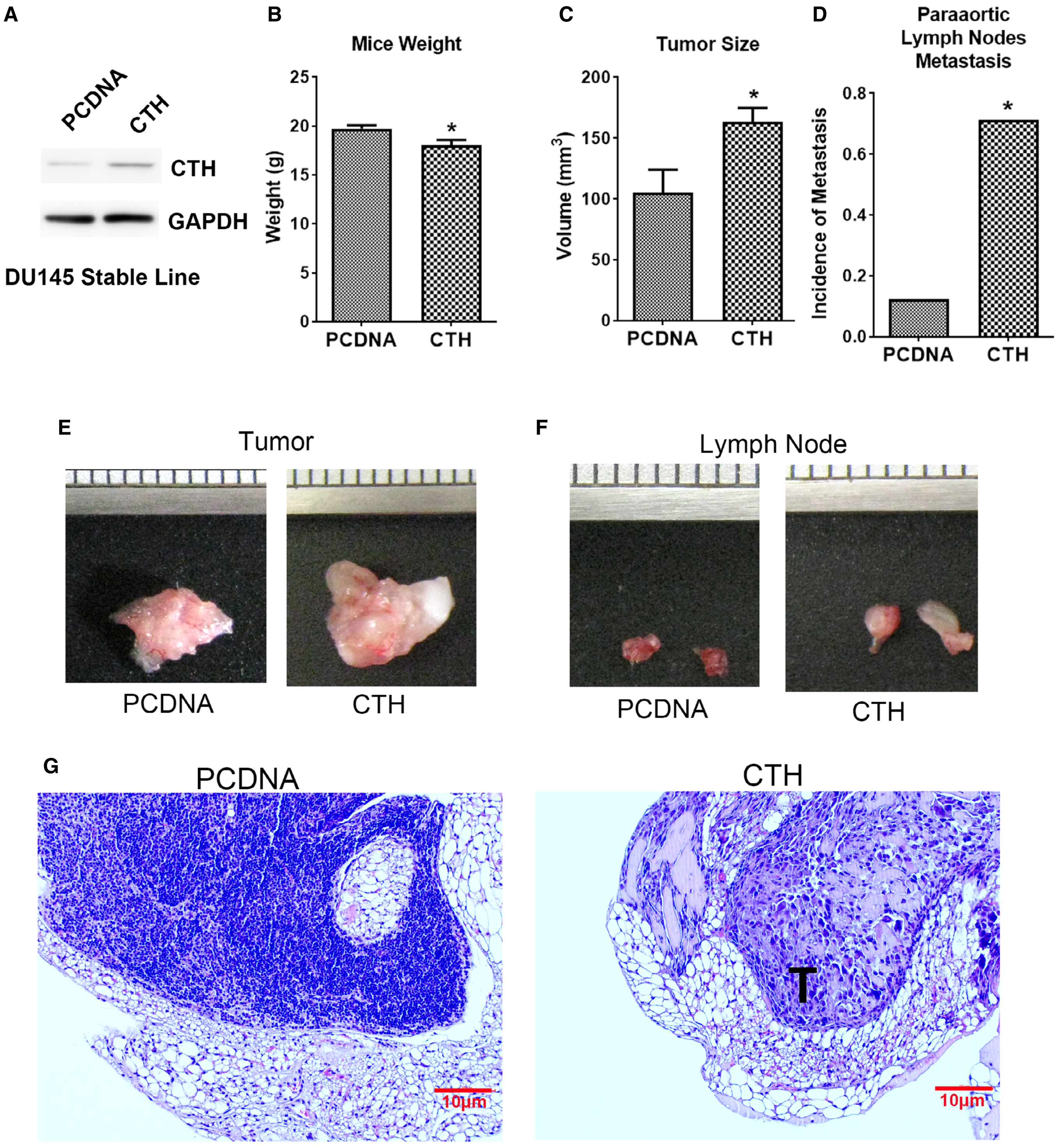

Figure EV5. Prostate orthotopic xenograft of DU145 cells with CTH overexpression induces tumor growth and incidence of paraaortic lymph node metastasis.

A Western blot analysis of the CTH expression in DU145 cells stably expressing PCDNA or PCDNA-CTH.

B–D 1×10^6 DU145 cells with PCDNA or PCDNA-CTH expression were orthotopically injected into the mouse prostate for 30 days. The mouse weight (B) and tumor volume (C) were compared. Incidences of paraaortic lymph node metastasis (D) are shown. Data in (B, C) are presented as means ± SEM (n = 7 or 8 mice per group). Student's t-test was used for the statistical analysis (*$P < 0.05$).

E, F Images of the orthotopic xenograft tumor (E) and paraaortic lymph nodes (F) of mice orthotopically implanted with DU145-PCDNA or CTH cells.

G Images show H&E staining of paraaortic lymph node sections. T: metastatic tumor cells (scale bar: 10 μm).

Source data are available online for this figure.

© 2019 The Authors
