# Supplementary figures and images for: Dysregulation of cystathionine γ‐lyase promotes prostate cancer progression and metastasis
Source: EMBO Rep. 2019 Aug 29;20(10):e45986. doi: 10.15252/embr.201845986 (PMC6776913; doi:10.15252/embr.201845986)

Figure EV1B

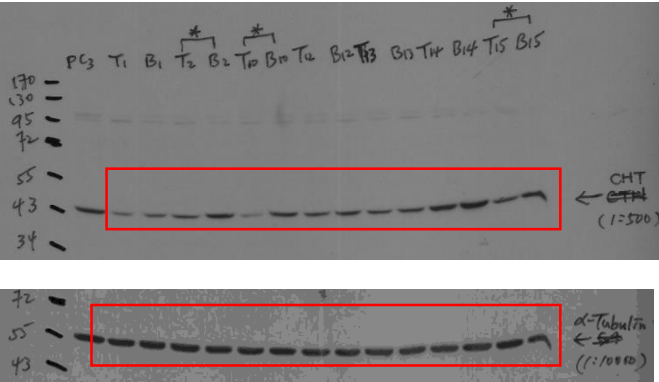

Figure EV1C

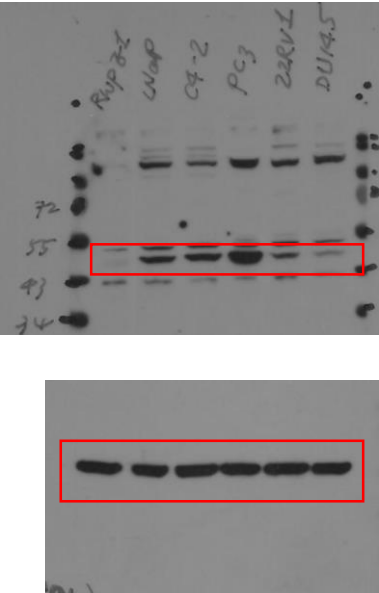

Figure EV1D

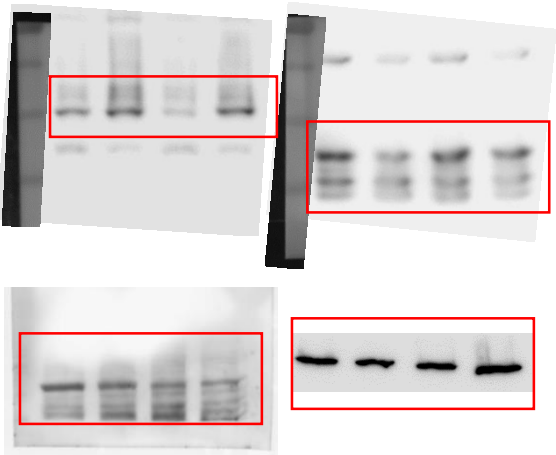

Supplement: Supplementary file 3 — Source Data for Expanded View [file EMBR-20-e45986-s007.zip › Source_Data_for_EV_Figures/Source_Data_for_FigEV1.pdf]

Figure EV2C

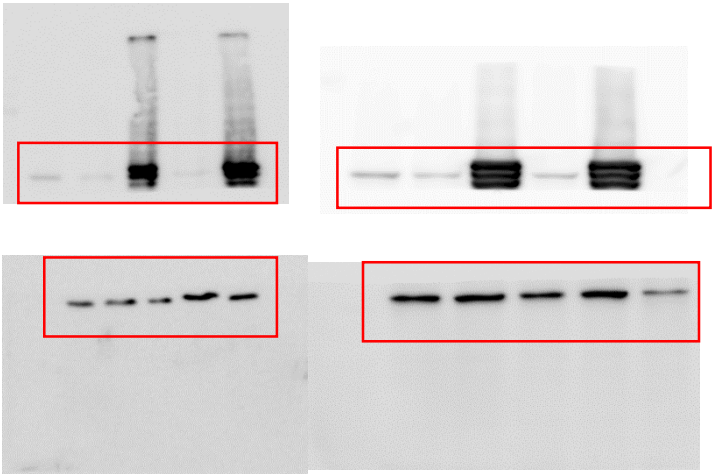

Supplement: Supplementary file 3 — Source Data for Expanded View [file EMBR-20-e45986-s007.zip › Source_Data_for_EV_Figures/Source_Data_for_FigEV2.pdf]

Figure EV3A

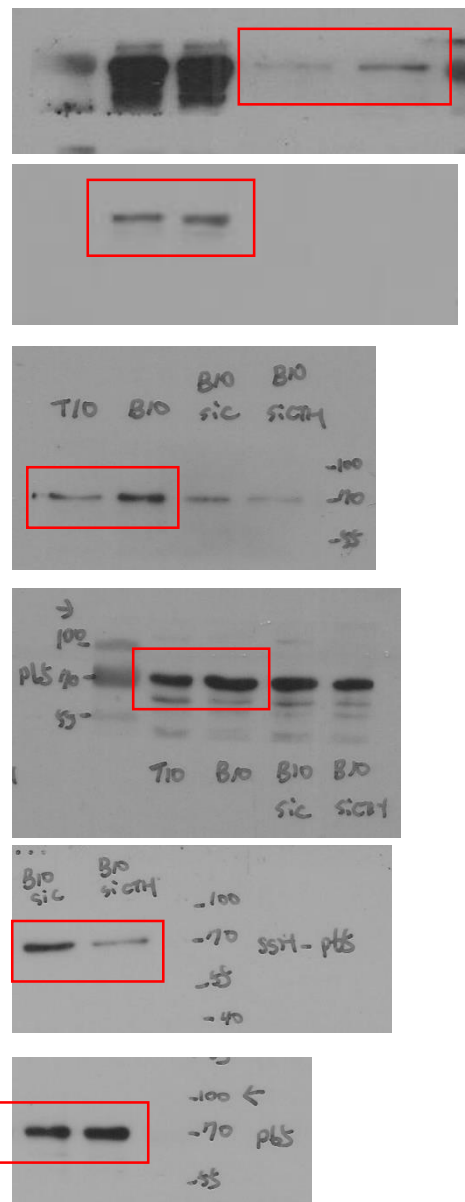

Figure EV3B

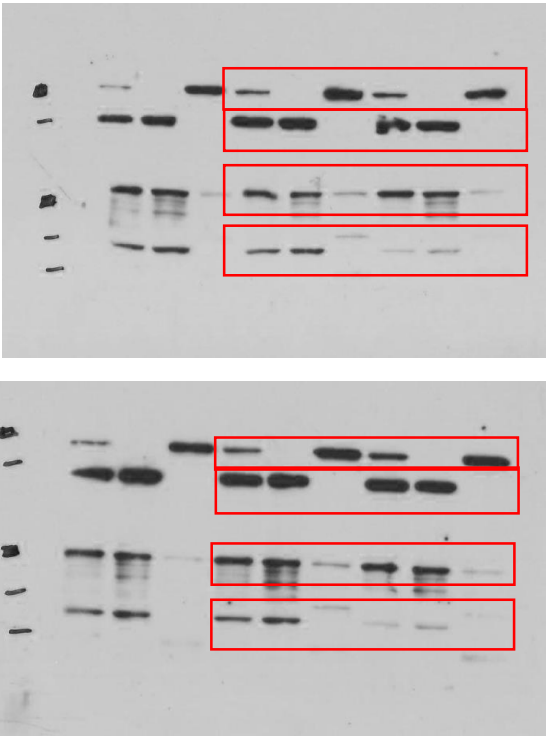

Figure EV3D

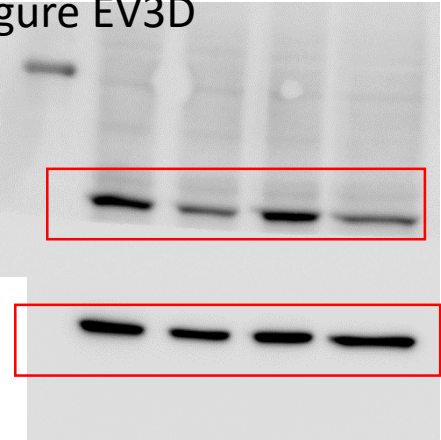

Figure EV3E

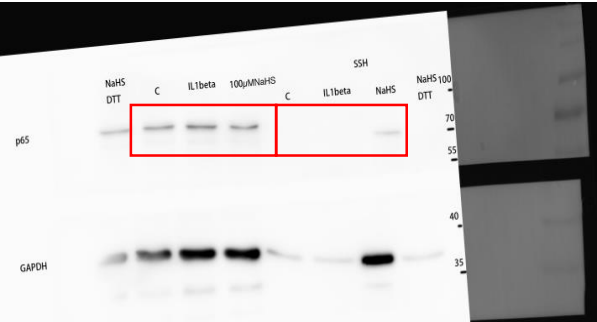

Supplement: Supplementary file 3 — Source Data for Expanded View [file EMBR-20-e45986-s007.zip › Source_Data_for_EV_Figures/Source_Data_for_FigEV3.pdf]

Figure EV4C

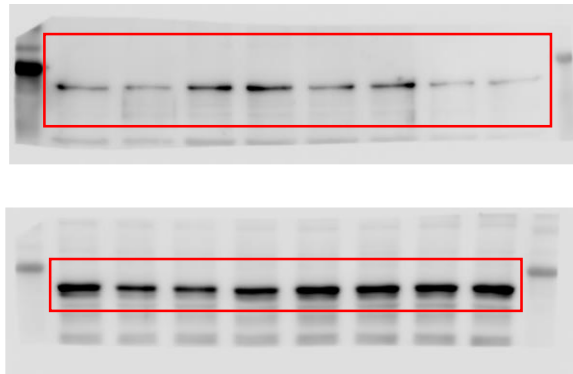

Figure EV4H

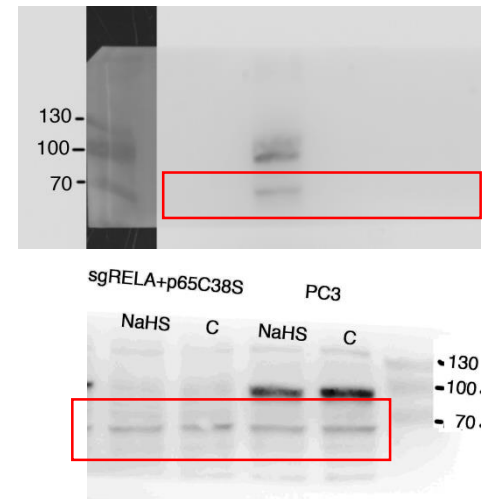

Supplement: Supplementary file 3 — Source Data for Expanded View [file EMBR-20-e45986-s007.zip › Source_Data_for_EV_Figures/Source_Data_for_Figure_EV4.pdf]

Figure EV5A

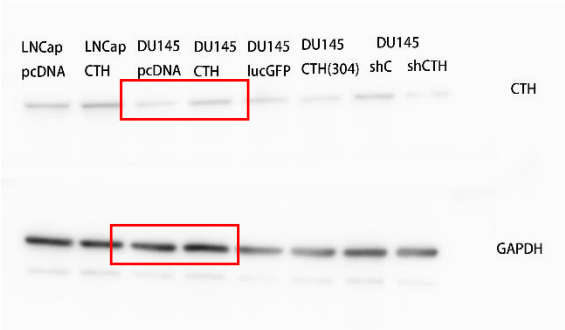

Supplement: Supplementary file 3 — Source Data for Expanded View [file EMBR-20-e45986-s007.zip › Source_Data_for_EV_Figures/Source_data_for_Figure_EV5.pdf]

Figure 1C

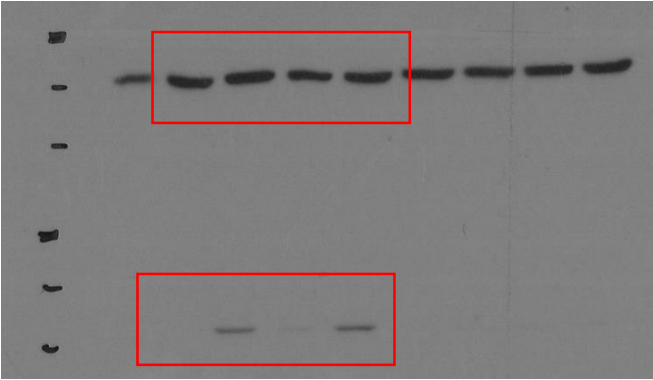

Supplement: Supplementary file 5 — Source Data for Figure 1 [file EMBR-20-e45986-s003.pdf]

Figure 2A

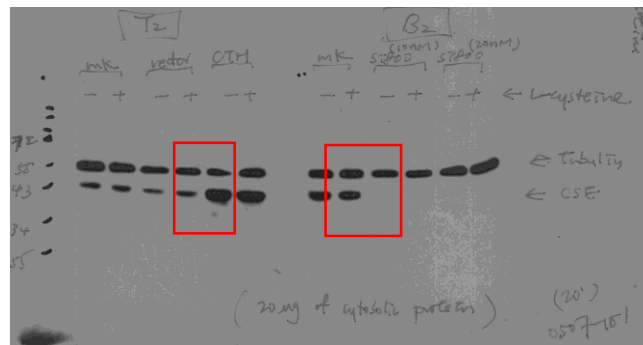

Figure 2E

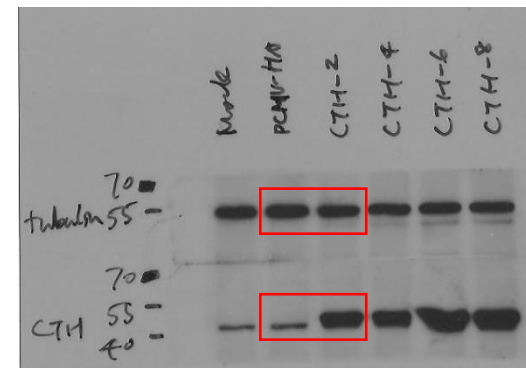

Supplement: Supplementary file 6 — Source Data for Figure 2 [file EMBR-20-e45986-s004.pdf]

Figure 4C

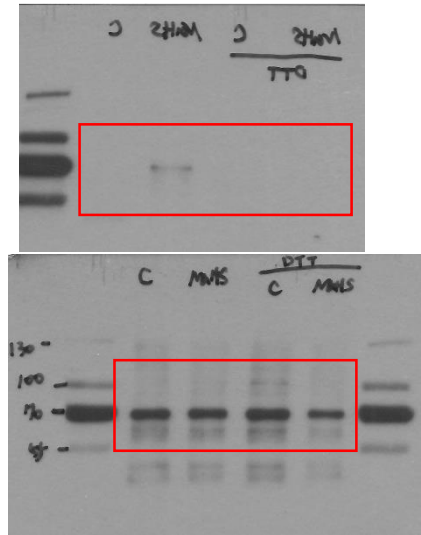

Figure 4G

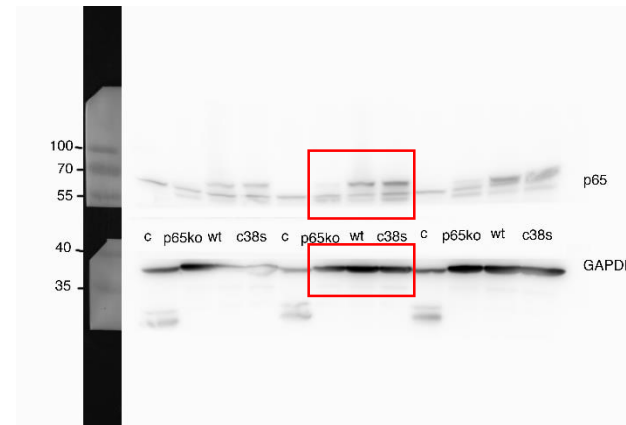

Supplement: Supplementary file 7 — Source Data for Figure 4 [file EMBR-20-e45986-s005.pdf]

Figure 5A

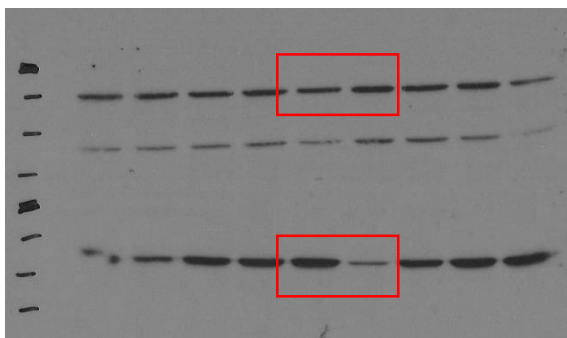

Supplement: Supplementary file 8 — Source Data for Figure 5 [file EMBR-20-e45986-s006.pdf]
